# Regulation of ABC transporters by sex steroids may explain differences in drug resistance between sexes

**DOI:** 10.1007/s13105-023-00957-1

**Published:** 2023-03-30

**Authors:** Rafael Mineiro, Cecília Santos, Isabel Gonçalves, Manuel Lemos, José Eduardo B. Cavaco, Telma Quintela

**Affiliations:** 1 CICS-UBI-Health Sciences Research Centre, University of Beira Interior, Av. Infante D. Henrique. 6200-506, Covilhã, Portugal; 2 UDI-IPG-Unidade de Investigação Para o Desenvolvimento Do Interior, Instituto Politécnico da Guarda, Guarda, Portugal

**Keywords:** ABC transporters, Sex steroids, Epithelial barriers, Sex differences

## Abstract

Drug efficacy is dependent on the pharmacokinetics and pharmacodynamics of therapeutic agents. Tight junctions, detoxification enzymes, and drug transporters, due to their localization on epithelial barriers, modulate the absorption, distribution, and the elimination of a drug. The epithelial barriers which control the pharmacokinetic processes are sex steroid hormone targets, and in this way, sex hormones may also control the drug transport across these barriers. Thus, sex steroids contribute to sex differences in drug resistance and have a relevant impact on the sex-related efficacy of many therapeutic drugs. As a consequence, for the further development and optimization of therapeutic strategies, the sex of the individuals must be taken into consideration. Here, we gather and discuss the evidence about the regulation of ATP-binding cassette transporters by sex steroids, and we also describe the signaling pathways by which sex steroids modulate ATP-binding cassette transporters expression, with a focus in the most important ATP-binding cassette transporters involved in multidrug resistance.

## Introduction

The efficacy of a drug is affected by the presence of epithelial barriers that limit the processes of absorption, distribution, and elimination. These epithelial barriers are highly selective and prevent the passage of many polar molecules. Two examples are the intestine barrier formed by the enterocytes and the blood–brain barrier (BBB) mainly formed by the brain capillary endothelial cells (BCEC). BBB is one of the most important barriers since it limits the passage of molecules from blood to the brain making difficult the development of drugs to treat brain diseases. At the molecular level, that function is conferred by tight junctions (TJ) between adjacent cells of the epithelial barriers which impair the paracellular movement of molecules, by detoxification enzymes, and efflux transporters [115], such as ATP-binding cassette (ABC) transporters. ABC transporters are expressed at barriers cells, where they actively extrude therapeutic drugs and xenobiotics out of the cells [128].

The different sex steroid background between sexes contributes to the differences in some physiological functions between men and women, for example, the differences between sexes in bone turnover [28], blood pressure [109], and also in neuroprotection [121]. This is supported by the wide distribution of sex steroid receptors in several organs. For example, they are present in vascular cells [51, 90, 112], the liver [4, 70, 98], the kidney [30, 99], the skin [120], the gastrointestinal tract [12], and brain cells [67, 83], expanding the list of target tissues beyond reproductive organs. The response to drug treatments differs between men and women due to relevant differences in drug pharmacokinetics [122, 125]. ABC transporters play a significant role in the absorption, distribution, and elimination of several drugs, thereby contributing to differences in responses to a large number of medications between men and women. Thus, the objective of the present review is to discuss the available evidences in the literature about the regulation of ABC transporters by sex steroids in several tissues and their possible involvement in drug resistance.

## ABC transporters

ABC transporters are a class of membrane proteins which couple the ATP hydrolysis to the extrusion of molecules against their electrochemical gradient [15]. In terms of structure, ABC transporters are composed by two transmembrane domains (TMDs) responsible for substrate translocation, and two intracellular nucleotide binding domains (NBDs) responsible for ATP hydrolysis [135]. The transport cycle of the ABC transporters is still a theme of debate [132]. Briefly, at an inward-facing conformation, the substrate binds to the TMDs from the cytoplasm or the inner leaflet of the lipidic bilayer. Two ATP molecules bind and induce the NBDs dimerization, leading to an occluded state. ATP molecules are hydrolyzed, and the ABC transporter adopts an outward-facing conformation allowing the substrate release. Finally, the ADP and Pi dissociate from the NBDs, and the exporter returns to the initial inward-facing conformation [15]. Moreover, ABC transporters can be composed by one or two protein subunits. In the human genome, there are 48 ABC coding genes distributed over 7 subfamilies (A-G) [106]. The most preponderant ABC transporters involved in multidrug resistance are MDR1 (or ABCB1), the first member of the ABCB subfamily, MRPs (1–9), from the ABCC subfamily, and BCRP (or ABCG2), the second member of the ABCG subfamily [94].

### Function and localization of ABC drug transporters

ABC drug transporters have the endogenous function of regulating the transport of various substances like nutrients, metabolites, bile salts, and many biologically relevant molecules [94]. Due to their function, ABC drug transporters are widely expressed in the epithelial cells of the organs involved in the absorption, distribution, and excretion of substances. Thus, ABC drug transporters are mainly present in the absorptive epitheliums of the intestine and lungs, in physiological barriers like BBB or blood–placenta barrier, and also in the liver and kidney, which function as excretory organs. The localization of the ABC drug transporters in the referred organs and tissues is summarized in Table 1. In addition, ABC drug transporters are also importantly expressed in tumoral tissues, where they play a pivotal role in chemotherapy resistance, which contributes to the growth and development of tumors [106].

**Table 1:** Human localization of ABC drug transporters in pharmacokinetics most relevant organs/tissue

Organ/tissue	Transporter	Localization	References
Intestine	MDR1	Enterocytes (apical)	[32, 53, 85]
	MRP1	Caco-2 cells (intracellular)	[24, 97]
	MRP2	Enterocytes (apical)	[85]
	MRP3	Caco-2 cells (basolateral)	[97]
	MRP4	Caco-2 cells (basolateral)	[81]
	BCRP	Enterocytes (apical)	[75, 82]
Lungs	MDR1	Brochiolar epithelium (apical)	[7]
		Ciliated cells of bronchiolar epithelium (apical)	[7, 11]
		Alveolar epithelium (apical)	[29]
		Alveolar type I cells (apical)	[11, 29]
Liver	MDR1	Hepatocytes (apical)	[45, 68]
		Cholagenocytes (apical)	[136]
	MRP2	Hepatocytes (apical)	[55, 68, 92, 119]
		Cholagenocytes (apical)	[18]
	MRP3	Hepatocytes (basolateral)	[56, 151]
		Cholagenocytes (basolateral)	[151]
	MRP4	Hepatocytes (basolateral)	[36, 104]
	MRP6	Hepatocytes (basolateral)	[118]
	BCRP	Hepatocytes (apical)	[68, 75]
Kidney	MDR1	Proximal tubule epithelial cells (apical)	[78]
	MRP2	Proximal tubule epithelial cells (apical)	[117]
	MRP4	Proximal tubule epithelial cells (apical)	[138]
	MRP6	Proximal tubule epithelial cells (basolateral)	[118]
	BCRP	Proximal tubule epithelial cells (apical)	[46]
Placenta	MDR1	Syncytiotrophoblasts (apical)	[2, 5, 58, 69, 89]
	MRP1	Syncytiotrophoblasts (basolateral)	[2, 5, 58, 89]
	MRP2	Syncytiotrophoblasts (apical)	[37, 79, 130]
	MRP3	Syncytiotrophoblasts (apical)	[130]
	MRP5	Syncytiotrophoblasts (basal)	[79]
	BCRP	Syncytiotrophoblasts (apical)	[38]
BBB	MDR1	BCEC (luminal)	[139]
	MRP1	BCEC (luminal)	[93]
	MRP4	BCEC (luminal)	[93]
	MRP5	BCEC (luminal)	[93]
	BCRP	BCEC (luminal)	[21, 25]
CP	MRP1	CPEC (basolateral)	[102]
	MRP4	CPEC (basolateral)	[61]

*BCEC*, blood capillary endothelial cells; *CPEC*, choroid plexus epithelial cells

Due to their expression and capacity of transporting therapeutic drugs, generally, ABC drug transporters can affect the efficacy of a drug in three ways: 1) by limiting the absorption; 2) when the drug has already reached the blood circulation, the presence of ABC transporters in intern physiological barriers limit the passage of the drug to its action site; and 3) the presence of ABC drug transporters in excretory organs facilitates the elimination of drugs and their metabolites [127].

#### Multidrug resistance protein 1 (MDR1)

The most well-characterized ABC drug transporter is MDR1 (or ABCB1), which was identified as an efflux pump in 1976 by Juliano and Ling [49]. MDR1 is capable of recognizing a vast array of structurally diverse substrates, and the majority of them are hydrophobic or amphipathic molecules [17]. For that reason, MDR1 is able to transport a great array of therapeutic drugs, like antibiotics, anticancer, and antiepileptic agents [35]. Aside from therapeutic drugs, MDR1 is also able to transport endogenous compounds and metabolites, like bile acids, bilirubin, and amyloid-β protein (Aβ) [35].

In absorptive organs, MDR1 is expressed in the apical membrane of the enterocytes [32, 53, 85] and on the apical surface of alveolar [29] and bronchial epithelium [7]. Hence, MDR1 can limit the bioavailability of orally and airway administrated drugs. In intern physiological barriers, MDR1 is located in the luminal section of the brain capillary endothelial cell membrane [139] and also on the apical surface of syncytiotrophoblasts in the placenta [2, 5, 58, 69, 89]. In the BBB, MDR1 plays the very important role of protecting the brain against xenobiotics and also in the removal of metabolites like Aβ into the blood stream. The evidence concerning the extrusion of the Aβ peptide by MDR1 in the BBB has already been reviewed [27]. Recent studies confirmed the MDR1 involvement in Aβ clearance from the brain, showing that the luminal accumulation of Aβ peptides in brain capillaries from wild-type mice was greater than in brain capillaries from *Mdr1* knock-out mice [13]. Another study showed that the inhibition of MDR1 with its specific inhibitor, PSC835, compromises the transport of Aβ peptide in mouse brain capillaries and in porcine BCEC [129]. However, besides these protective roles, MDR1 can limit the drug passage to the brain tissue, which is difficult in the development of drugs for brain diseases. In the placenta, MDR1 protects the fetus from mother-born noxious compounds. The evidence of the MDR1 protective role in the placenta was extensively reviewed by Joshi et al. [48]. Finally, MDR1 is also present in the membrane of the hepatocytes facing the bile canaliculus (apical) [45, 68] and in the apical membrane of the proximal tubule epithelial cells [78]. Thus, MDR1 contributes to the elimination of substances through the bile and also participates in the tubular secretion process.

#### Multidrug resistance-associated proteins (MRPs)

MRPs are members of the ABCC subfamily [84] and are also capable of transporting a wide range of endogenous substrates and therapeutic agents, like antiretroviral and anticancer drugs [35]. MRP1, the most well-characterized MRP, has a considerable range of endogenous substrates and, for example, is capable of transporting leukotrienes [62], folates [149], and many other endogenous substances along with several therapeutic agents [35]. Another example is MRP4, which, in addition to therapeutic drugs, is capable of transporting substances like cAMP [22] and prostaglandins [103].

Among the MRPs expressed in the intestine, only MRP2 was found to be localized on the apical side of the membrane of the enterocytes [85]. Data from the Caco-2 cell line reported that MRP1 has an intracellular localization [24, 97] and that MRP3 [24, 97] and MRP4 have a basolateral localization [81]. So, only MRP2 is able to pump drugs back into the intestinal lumen. Regarding the brain barriers, in the BBB, MRP1, MRP4, and MRP5 are localized on the luminal side of the membrane of the brain capillary endothelial cells [93]. In the blood cerebrospinal fluid barrier (BCSFB), MRP1 is localized on the basolateral membrane of the choroid plexus epithelial cells (CPEC) [102]. Still in the BCSFB, MRP4 is also expressed on the basolateral membrane of the CPEC [61]. So, MRPs like MDR1 also limit the passage of drugs to the brain. In the placenta, MRP2 and MRP3 were on the apical side [37, 79, 130], while MRP5 and MRP1 are localized on the basolateral side of the membrane of the syncytiotrophoblasts [2, 5, 38, 58, 89]. Here, only MRP2 and MRP3 may participate in the fetus protection from deleterious substances derived from maternal blood. In the liver, MRP2 was identified in the apical membrane of the hepatocytes [55, 68, 93, 119]. On the other hand, MRP3 and MRP4 are present in the basolateral side [36, 56, 104, 151]. In the kidney, MRP2 and MRP4, like MDR1, were shown to be located in the apical side of the proximal tubule epithelial cells [117]. Thus, MRP2 and MRP4 promote the elimination of substances in the kidney through tubular secretion.

#### Breast cancer resistance protein (BCRP)

BCRP was first identified in 1998 by Doyle et al. in MCF7 cells [26]. Contrarily to the previous ABC transporters, BCRP is considered a half-transporter since it is composed of one NBD and one TMD and needs to homodimerize to form a functional transporter in the plasma membrane [116]. BCRP is known for its role in pharmacoresistance. Like MDR1, BCRP is also capable of transporting a wide array of hydrophobic and amphipathic drugs from a wide range of therapeutic agents. BCRP can also transport a few metabolites like estrone-3-sulphate and uric acid [35, 116].

Regarding absorptive epitheliums, BCRP is expressed in the intestine and it is localized in the apical membrane of enterocytes [75, 82]. Thus, BCRP can limit the absorption of orally administrated drugs. In the intern physiological barriers, BCRP is expressed in the luminal side of the BCEC [21, 25] where it contributes to brain pharmacoresistance. BCRP is also expressed in the blood–placenta barrier, more precisely in the apical surface of the syncytiotrophoblasts [38]. Moreover, BCRP is present in the apical side of the membrane of proximal tubule epithelial cells, in the kidney, and also in the apical surface of the hepatocytes [46]. Thus, BCRP also contributes to the elimination of substances through the bile and also by contributing to the tubular secretion process in the kidneys.

## Regulation of ABC drug transporters by sex hormones

### Multidrug resistance protein 1 (MDR1)

A few studies have been published demonstrating the differential MDR1 expression between sexes (Table 2). In mouse kidneys, Kanado et al. showed that MDR1 has a sex-dependent expression. MDR1 protein expression showed approximately 1.5-fold higher expression in females than in males [50]. In rodents, there are two isoforms of MDR1 encoded by the *Mdr1a* and *Mdr1b* genes. A study with C57BL/6 mice reported that *Mdr1b* gene has a gender-dependent expression in the kidney, brain, and lungs. In the kidney and lungs, *Mdr1b* gene has a higher expression in females than in males, while in the brain, the expression was higher in males. Additionally, the expression of *Mdr1a* gene in the kidney was higher in females when compared to male mice [23]. In the BCSFB, *Mdr1a* and *Mdr1b* genes did not show differences in expression between male and female rats, and their expression is not influenced by female or male sex hormone background [100, 101, 114].

**Table 2:**
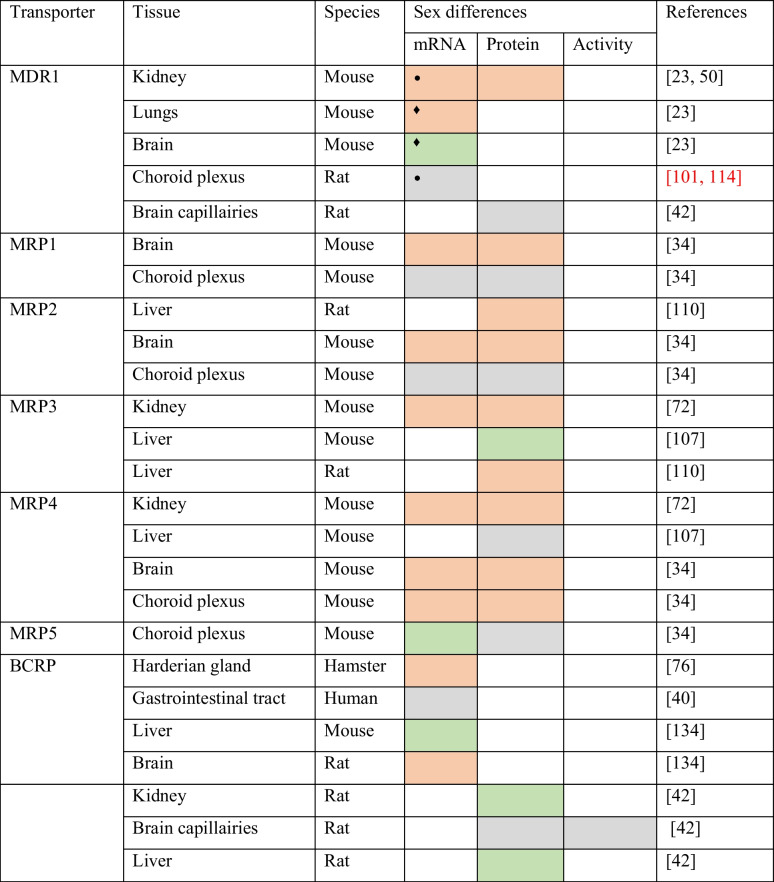
Sex differences in ABC drug transporters expression

Higher expression in females when compared with males

Higher expression in males when compared with females

No significant differences between sexes

^•^Mdr1a and Mdr1b

^♦^Mdr1b

The evidence of sex differences in MDR1 expression signals a possible regulation by sex steroid hormones. The evidence about the regulation of MDR1 by sex hormones and sex hormone receptor modulators is summarized in Table 3.

**Table 3:**
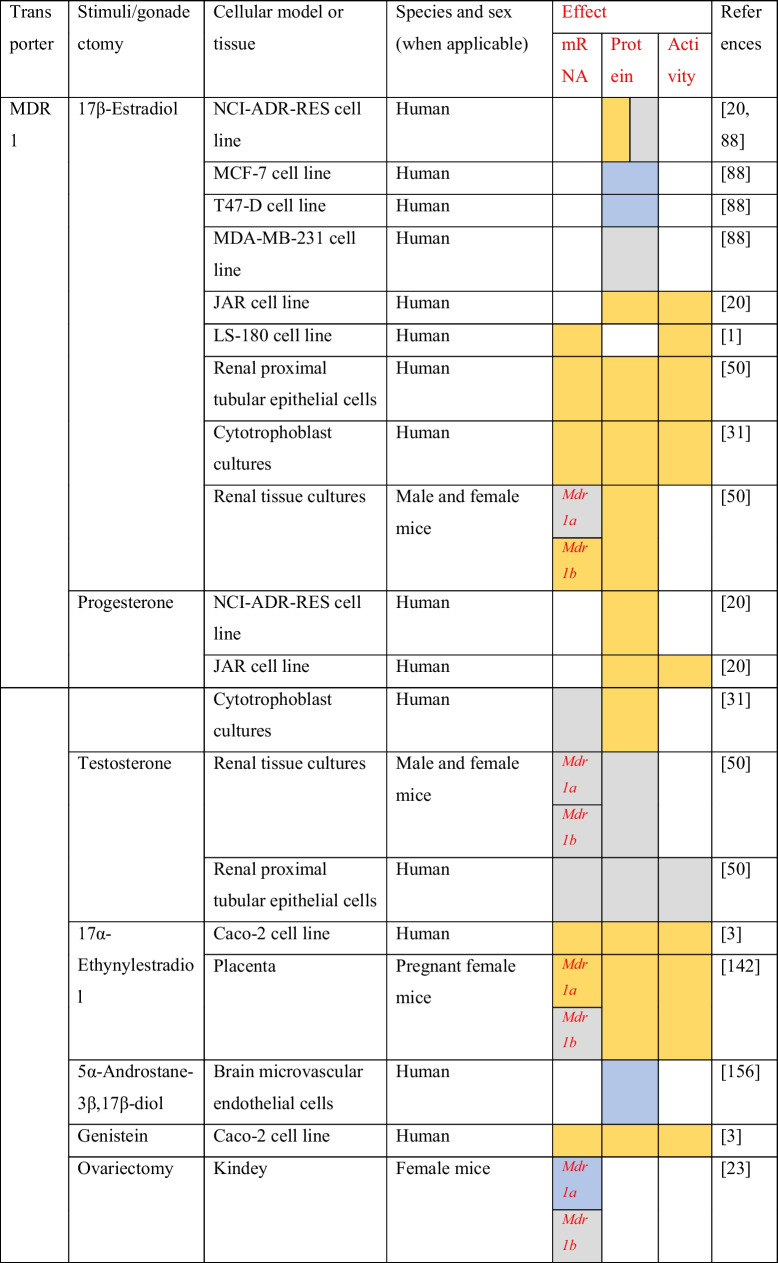

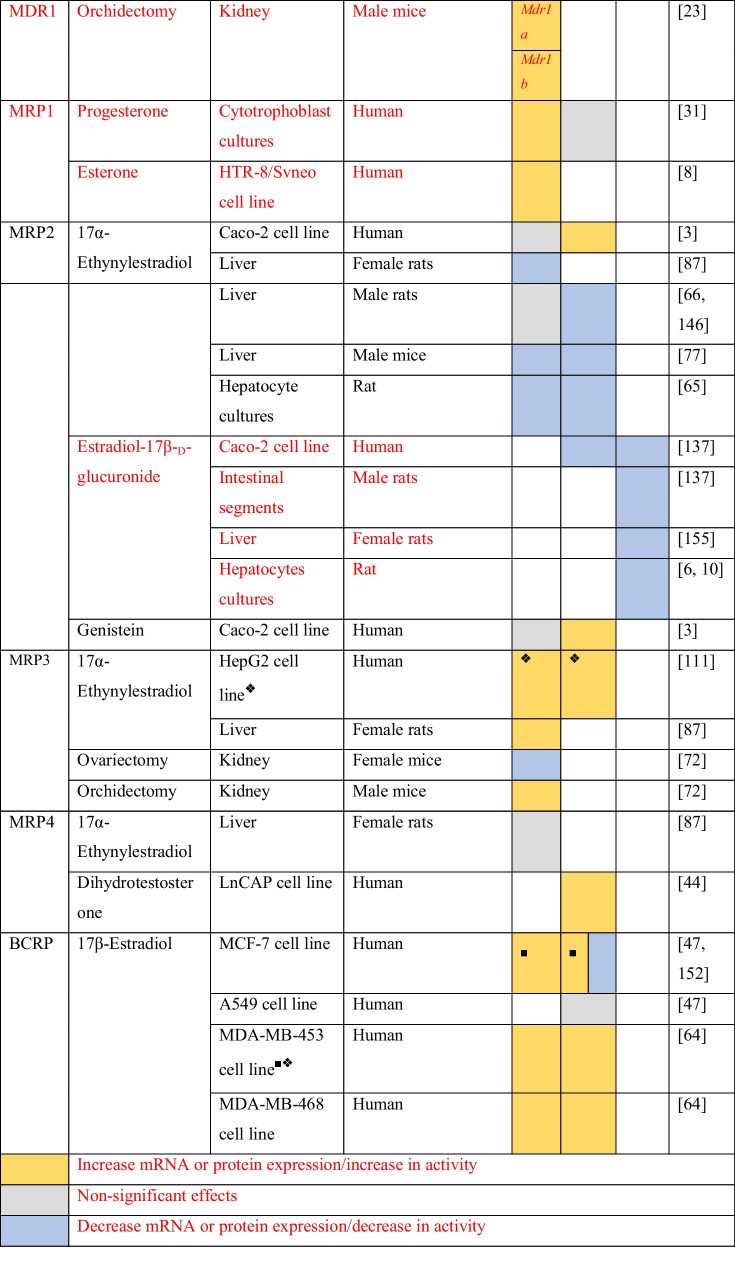

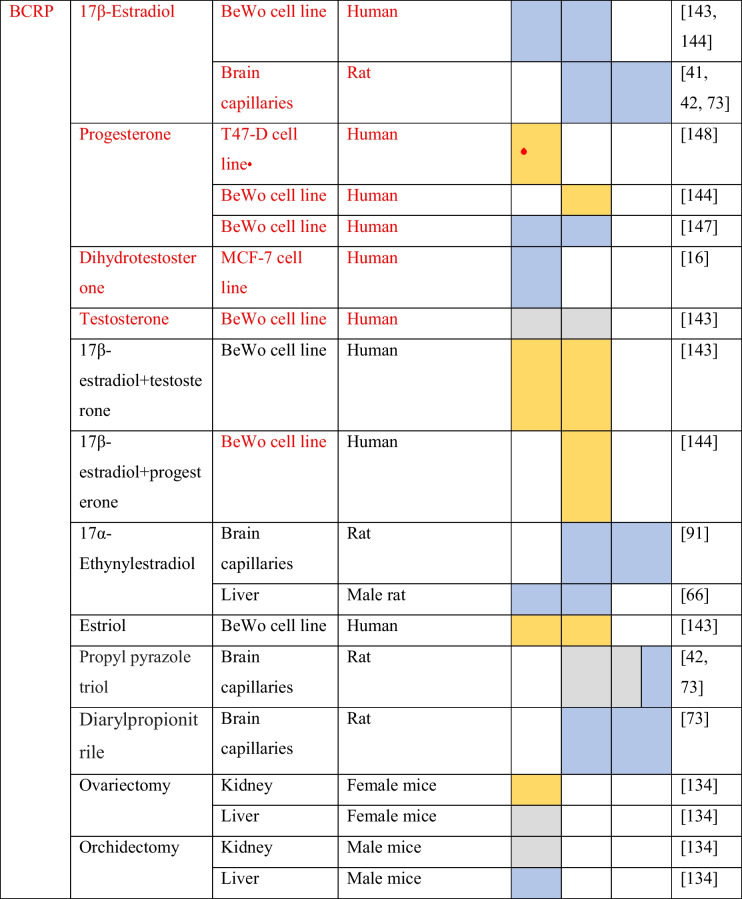
Regulation of ABC drug transporters by sex steroids and sex hormone receptor modulators

Increase mRNA or protein expression/increase in activity

Non-significant effects

Decrease mRNA or protein expression/decrease in activity

^❖^Cells were transfected with plasmids containing the Erα/β gene

^*■*^Cells were transfected with a Bcrp gene containing plasmid

Luciferase assay

Pregnant female C75BL mice injected for 4 days with 17α-ethynylestradiol, an agonist of estrogen receptors, led to the upregulation of MDR1 mRNA (*Mdr1a*) and protein in the placenta. On the other hand, 17α-ethynylestradiol does not show any significant effects in the expression of *Mdr1b* gene on the placenta. Digoxin is a known MDR1 substrate and is specifically transported by MDR1 across the placenta. The authors showed that the administration of 17α-ethynylestradiol decreased the digoxin transport from the mother to fetus [143]. In the kidneys, 17β-estradiol (E2) upregulated the expression of *Mdr1b* gene and MDR1 protein in mouse renal tissue cultures [50]. In addition, Cui et al. showed that after gonadectomy, *Mdr1a* gene expression increased in male and decreased in female mouse kidneys [23].

The evidence about the sex steroid regulation of MDR1 expression in humans comes from in vitro studies. In NCI-ADR-RES and placental JAR cell lines, E2 leads to an upregulation of MDR1 protein levels in a concentration-dependent manner. In the same study was also shown that E2 stimuli lead to a decrease in the saquinavir uptake in JAR cell line. This effect was abrogated in the presence of verapamil, a MDR1 inhibitor [20]. In human cytotrophoblasts, treatment with E2 upregulated MDR1 protein and mRNA expression. The treatment with E2 also caused a decrease in the intracellular accumulation of digoxin [31]. In addition, the treatment of the colon adenocarcinoma LS-180 cells with E2 leads to an upregulation of *Mdr1* gene expression. The same study also assessed the effect of E2 on MDR1 where E2 increased the MDR1 activity in cells incubated with rhodamine 123 (rho 123, a MDR1 substrate) [1]. In the human renal proximal tubular epithelial cells, the treatment with E2 increased the mRNA and protein MDR1 expression. The effects of E2 on MDR1 activity were also investigated and, for that, after the treatment with E2, human renal proximal tubular epithelial cells were incubated with digoxin in the presence or absence of PSS833, a MDR1 inhibitor. They observed that E2 increased MDR1 activity by approximately 0.5-fold [50].

In a mechanistic perspective, there is data demonstrating that MDR1 is regulated by sex steroids via both nuclear estrogen receptors (ER). In Caco-2 cells, which only express ERβ [12], the administration of 17α-ethinylestradiol increases mRNA and protein MDR1 expressions. The pre-treatment of Caco-2 cells with ICI 182,780 (ICI), an estrogen receptor antagonist, reverted the upregulation of MDR1 induced by 17α-ethinylestradiol. The authors also demonstrated that the stimulation with 17α-ethinylestradiol lead to a decrease in Rh123 uptake. The presence of verapamil reverted the effects induced by 17α-ethinylestradiol [3]. Another study with four breast cancer cell lines, two Erα^+^ positive (MCF-7 and T47-D) and two ER^−^ negative (MDA-MB-231 and NCI/ADR-RES), revealed that E2 only decreased the expression of MDR1 protein levels in ERα-positive cell lines [88]. Estrogens may regulate MDR1 expression through genomic signaling pathways. However, there is no data clarifying the involvement of ERs binding to the *Mdr1* gene, or the interaction of ERs with other transcription factors. These data suggest that ERβ upregulates the *Mdr1* expression and ERα downregulates this gene. However, a study where human brain microvascular endothelial cells (hBMEC) were treated with 5α-androstane-3β,17β-diol demonstrated a downregulation of MDR1 (protein) expression. Nonetheless, the pre-treatment with ICI reverted the effect of 5α-androstane-3β,17β-diol [154]. 5α-androstane-3β,17β-diol is an androgen metabolite which is a total agonist for ERβ and binds with low affinity to ERα. In BBB, ERβ shows a higher expression than ERα [73]. Thus, ERβ may be able to modulate MDR1 expression in the BBB. In Caco-2 cells, the effect was distinct, and the ERβ activation led to an upregulation of MDR1, contrarily to the downregulation observed in hBMEC. A possibly explanation may reside in different interactions of ERβ with other transcription factors and in ERβ recruitment of different chromatin coregulators in these two cell types, what highlights the hypothesis of a tissue-dependent regulation by estrogens.

According to the presented data, it is evident that, at least, estrogens regulate MDR1 expression. For progesterone and testosterone, the evidence is limited, although a few studies report that progesterone upregulates MDR1 (mRNA and protein) expression in human cytotrophoblasts [31] and in NCI-ADR-RES and placental JAR cell lines [20]. Also, in JAR cell line, P4 stimuli lead to a decrease in the saquinavir uptake and the presence of verapamil reverted the effect [20]. On the other hand, the evidence about the testosterone regulation of MDR1 comes from studies in rodents or with rodents’ cell cultures. It was shown that testosterone has no effect on MDR1 protein expression in mouse renal tissue cultures and in human renal proximal tubular epithelial cells. In human renal proximal tubular epithelial cells, it was also showed that testosterone induced no effects in MDR1 activity [50]. In contrast, in vivo testosterone results are not in line with those observed for in vitro experiments. Gonadectomy of male mice increase *Mdr1a* and *Mdr1b* gene expressions in the kidney, and the subsequent hormone replacement with dihydrotestosterone (DHT) induced *Mrd1a* and *Mdr1b* expression levels to return to those observed in control mice [23]. In Wistar rats administrated with the excipients, Gremopher RH, Poloxamer 188, and Tween 80 were observed a variation in the testosterone plasma concentration and in the MDR1 protein expression in jejunal segments [74].

### Multidrug resistance-associated proteins (MRPs)

As for MDR1, there are also some studies reporting sex differences in MRP expression (Table 2). MRP3 and MRP4 (protein and mRNA) have a sex-related expression in mouse kidneys with a greater expression in females [72]. In the liver, higher levels of MRP3 protein expression were reported in female when compared to male mice. The expression of MRP4 was only higher in the female liver in mice which were submitted to a fasting period [107]. A study with Sprague–Dawley rats showed that MRP2 and MRP3 (mRNA and protein) have higher expression levels in female livers when compared to male rats [110]. In the CP of C57BL/6 mice, MRP4 showed higher mRNA and protein expression levels in females when compared to male mice. Contrarily, *Mrp5* gene showed higher mRNA expression levels in the CP of male mice. For MRP1 and MRP2, no differences were observed in their expression levels between sexes [34]. Santos and co-workers found that the *Mrp1* gene has a higher expression level in the CP of male rats when compared with female rats [114]. Furthermore, in vitro studies performed by others, where mouse CP was incubated with fluo-cAMP (a known substrate for MRP4) revealed a higher fluorescence intensity in the vascular/perivascular spaces of female compared with male CP. The same study also revealed a 40% fluorescence reduction in the vascular/perivascular spaces in the CP of female *Mrp4* knock-out mice when compared to the CP of wild-type female mice [34].

Most of the evidence on the regulation of MRPs by sex steroids is summarized in Table 3. In rodents, the treatment of rat hepatocytes with 17α-ethynylestradiol decreased the expression of MRP2 (mRNA and protein) [65]. These results are corroborated by in vivo experiments. Studies with male Sprague–Dawley and Wistar rats showed that the administration of 17α-ethynylestradiol contributes to the downregulation of MRP2 protein expression in the liver [66, 144]. Male C57BL/6 mice (8–9 weeks) treated with 17α-ethynylestradiol also showed a reduced mRNA and protein MRP2 expression in the liver when compared to control mice [77]. In rat hepatocytes, estradiol-17β-_D_-glucuronide, a 17β-estradiol endogenous metabolite, decreased the activity of MRP2, and also led to MRP2 internalization [6, 10]. A study performed by Zucchetti et al*.*, where female Wistar rats were perfused with 1-chloro-2,4-dinitrobenzene (CDNB), a MRP2 substrate, showed that estradiol-17β-_D_-glucuronide reduced the elimination of the CDNB intrahepatic metabolite dinitrophenyl-gluthatione (DNP-G) in the bile for approximately 35% of its basal levels [153]. In rat intestinal segments, estradiol-17β-_D_-glucuronide also decreased the activity and expression (protein) of MRP2 [137]. The treatment of male rats with dehydroepiandrosterone (DHEA), a hormone precursor capable of binding to many nuclear receptors including ERs and androgen receptor (AR) [19], decreased the expression of MRP2 (protein) in the liver of male rats, and increased MRP3 (protein) expression in the liver of female rats [110].

In human colorectal cancer Caco-2 cells, estradiol-17β-_D_-glucuronide decreased the activity and induced the internalization of MRP2 [137]. On the contrary, an increase in MRP2 protein expression was observed after treatment of Caco-2 cells with 17α-ethynylestradiol [3]. This difference may be due to the fact that in the first study they access the protein expression in the brush border membrane, and in the other study, the authors quantify the total membrane protein. Tochetti et al. also showed that estradiol-17β-_D_-glucuronide increase the expression of MRP2 in intracellular membrane fractions. Also, in the same cell line, the treatment with genistein, a full agonist of ERβ, increased the expression of MRP2 (mRNA and protein) [3].

**Fig. 1:**
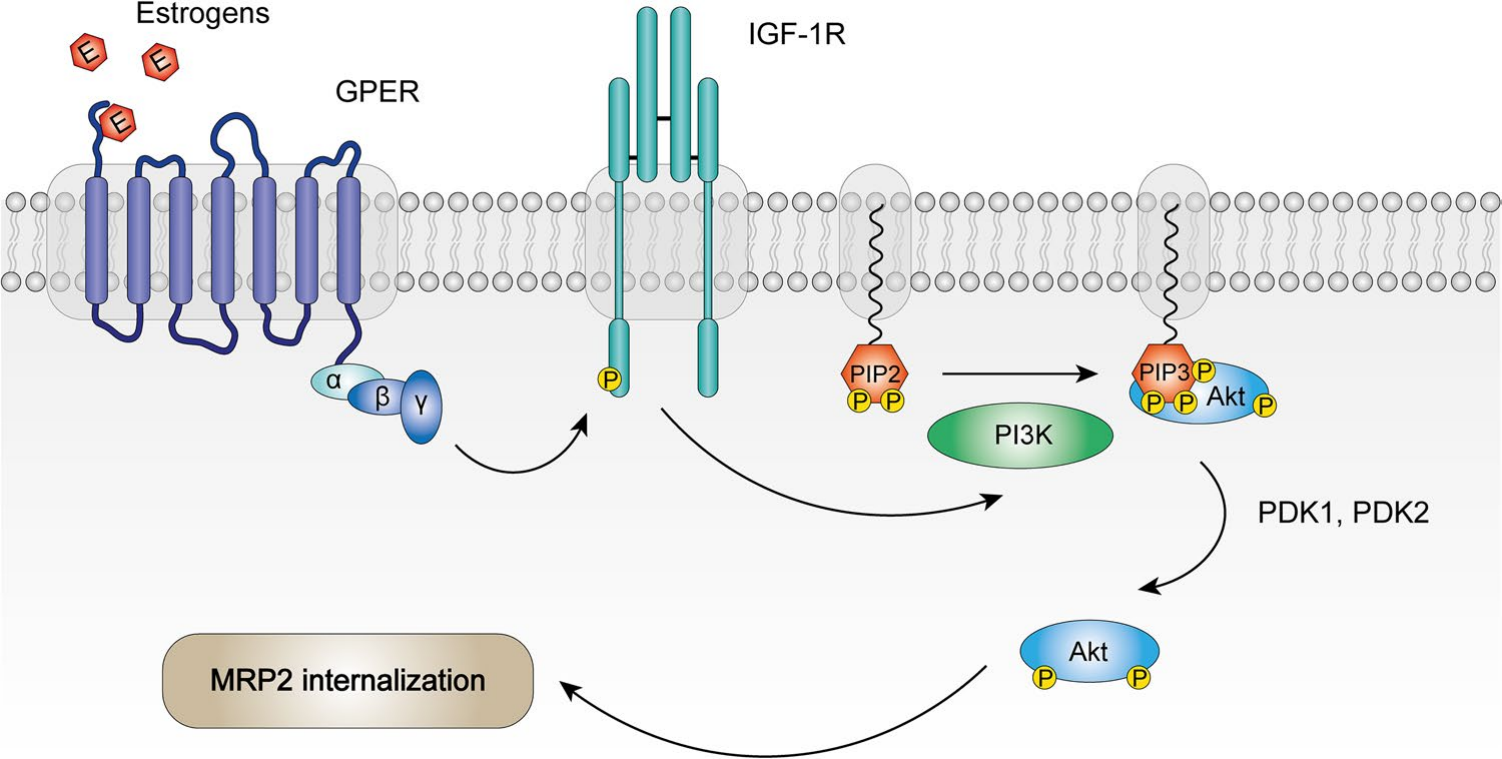
MRP2 regulation by estrogens. The activation of GPER leads to the transactivation of insulin growth factor 1 receptor (IGF-1R). IGF-1R is a tyrosine kinase receptor. In its turn, IGF-1R leads to the activation of PI3K which converts PIP2 in PIP3, providing an anchorage point for Akt. The anchorage of Akt enables its activation by phosphorylation. The active Akt lead to MRP2 membrane internalization

At the signaling level, in the literature, a few studies have tried to uncover the signaling pathways involved in MRP2 regulation by estrogens in the liver. The pre-treatment of rat hepatocytes with G15, a GPER1 inhibitor, reverted the downregulation of MRP2 activity induced by estradiol-17β-_D_-glucuronide. Additional experiments revealed that estradiol-17β-_D_-glucuronide promotes the internalization of MRP2, which was abrogated in the presence of Tyrphostin AG 1024 (TYR), an IGf-1R inhibitor. Also, IGf-1R knockdown, abrogated the downregulation of MRP2 activity induced by estradiol-17β-_D_-glucuronide. In the first instance, this data shows that GPER and IGf-1R are involved in MRP2 regulation by estrogens. In the same study, the pre-treatment with wortmannin, a PI3K inhibitor, TYR and with both compounds, partially abrogated the decrease in MRP2 activity induced by estradiol-17β-_D_-glucuronide. Thus, IGF-1R and PI3K are involved in the same pathway [6]. Another study with a different estrogenic compound reported that the administration of 17α-ethynylestradiol in the presence of LY294002, a PI3K inhibitor, reverted the effect induced by 17α-ethynylestradiol on MRP2 protein expression in rat’s liver [144]. This result reinforces the involvement of PI3K in MRP2 regulation by estrogens. Further experiments showed that G15 decreased IGf-1R activation induced by estradiol-17β-_D_-glucuronide. So, the activation of GPER precedes the activation of IGf-1R. Also, the pre-treatment with TYR prevented Akt activation by estradiol-17β-_D_-glucuronide [6]. In summary, the signaling pathway involves the activation of GPER, which in turn activates IGf-1R. The IGF-1R through the PI3K/Akt pathway induces MRP2 internalization and a consequent decrease in its activity (Fig. 1). This pathway involves the crosstalk with GPER and IGF-1R, a tyrosine kinase receptor (RTK). The crosstalk of GPER with epidermal growth factor receptor (EGFR), another tyrosine kinase receptor, was also reported [96]. In addition to the IGF-1R/PI3K/Akt pathway, estrogens may regulate MRP2 through other signaling pathways, as TYR only showed a partial impediment of MRP2 activity downregulation by 17α-ethynylestradiol. ICI also partially abrogated the effect of 17α-ethynylestradiol on MRP2 activity. This result indicates that ER nuclear receptors are also involved in the regulation of MRP2 by estrogens [6]. In pregnancy, the IGF-1R/PI3K/Akt pathway may be preponderant because the studies performed to unravel the MRP2 regulation by this molecular pathway were conducted in order to uncover the molecular causes of intrahepatic cholestasis. Intrahepatic cholestasis is a liver disease that is common in pregnancy, in which high levels of estrogens seem to be one of the factors involved in its development.

**Fig. 2:**
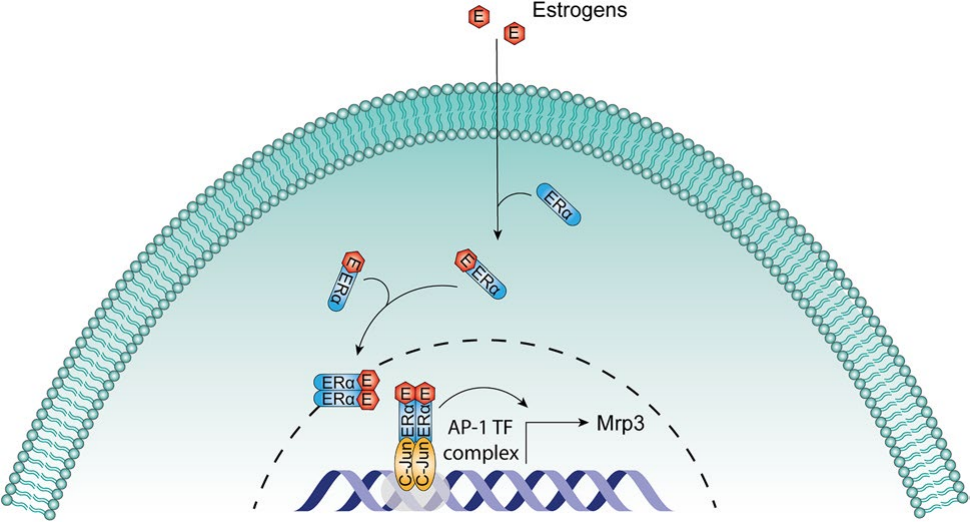
*Mrp3* regulation by ERα. *Mrp3* gene transcription is regulated by an estrogen indirect genomic signaling mechanism. In *Mrp3* gene, which lack an estrogen response element (ERE), ERα promotes the gene transcription by interacting with c-Jun, a member of AP-1 complex, which function as a transcription factor

There is also evidence about the signaling pathways involved in MRP3 regulation by estrogens. In the HepG2 cell line transfected with a plasmid containing ERα gene under a CMV promotor, the treatment with 17α-ethynylestradiol upregulated mRNA and protein MRP3 expression. Further experiments using the same cell line showed that silencing *c-jun* prevented the upregulation of MRP3 (protein) by 17α-ethynylestradiol. In addition, immunoprecipitation analysis showed that this estrogenic compound also promotes the interaction of ERα and c-Jun. This interaction is concordant with an in silico analysis, showing the absence of estrogen response elements in the *Mrp3* gene promotor [111]. Then, the putative signaling pathway of MRP3 expression regulation by estrogens involves an indirect genomic mechanism. After the ligand binding and ERα dimerization, ER complex interacts with c-Jun, which is a member of the AP-1 complex, to drive *Mrp3* gene expression (Fig. 2).

Furthermore, gonadectomy increased *Mrp3* gene expression in the kidneys of male mice when compared to intact males. Contrarily, in female mice, ovariectomy decreased *Mrp3* gene expression in the kidneys in comparison with intact female mice [72]. 17α-Ethynylestradiol increased *Mrp3* mRNA levels in female rat hepatocytes [87]. In terms of signaling, the outcome was similar to that observed for HepG2 cells treated with 17α-ethynylestradiol. Then, it is likely that in mouse kidneys and hepatocytes, estrogens also drive *Mrp3* expression through the indirect genomic pathway previously reported for HepG2 cells.

Taking into account these data, it is clear that estrogens regulate MRP2 and MRP3 expressions. Generally, estrogens downregulate MRP2 and upregulate MRP3. These data probably explain why MRP3 has a greater expression in female mouse kidney [72] and female rodents’ livers when compared to males. However, the fact that estrogens downregulate MRP2 cannot explain why MRP2 expression is higher in female rat livers than in the livers of male rats [110]. This hypothesis is supported by a tissue-dependent regulation.

The evidence concerning the regulation of MRP1 and MRP4 by the sex hormones is very limited, only a few studies were reported. In human cytotrophoblast primary cultures, treatment with progesterone increased *Mrp1* gene expression [31]. The treatment of immortalized trophoblast cells (HTR-8/SVneo) with estrone (E1) upregulated *Mrp1* gene expression [8]. In LNCaP cells, stimulus with DHT increased MRP4 protein expression, while in the same cell line transfected with luciferase reporter plasmids cloned with two fragments of *Mrp4* promotor, DHT did not exert any significant effect on luciferase fluorescence [44]. In rodents, 17α-ethynylestradiol has no significant effects on *Mrp4* expression in female rat’s hepatocytes [87]. Besides the lack of data, this cannot explain why MRP4 has a higher expression in female mouse’s CP and kidney when compared to male mice [34, 72].

### Breast cancer resistance protein (BCRP/ABCG2)

Studies on the expression analysis of BCRP have shown sex-specific expression in some tissues (Table 2). The expression of *Bcrp* gene in the Harderian gland is higher in females when compared to male hamsters [76]. The expression of *Bcrp* gene through the human gastrointestinal tract is similar between males and females [40]. In addition, sex differences in the rat brain and in the mouse liver have been reported. In the rat brain, BCRP mRNA expression is higher in females when compared to males. Contrarily, in the mouse liver, the expression of the BCRP (mRNA) is higher in males. [134]. This indicates a possible regulation of BCRP by sex hormones (Table 3).

E2 treatment of MCF-7 cells transfected with a plasmid containing the *Bcrp* gene upregulated mRNA and protein BCRP expression. Tamoxifene, an antiestrogenic compound, reverted the upregulation induced by E2. Electrophoretic mobility shift assays performed with nuclear extracts of MCF-7 cells, which are ERα positive, in parallel with nuclear extracts of MDA-MB-231 ERα-negative cells, revealed a band shift resulting from ERα binding to the probe containing *Bcrp* gene promotor, not observed in MDA-MB-231 cells [150]. In fact, E2 treatment of MCF-7 cells downregulated BCRP protein expression. Contrarily, in A549 cells (negative for estrogen receptors), E2 does not significantly affect BCRP protein expression [47]. These controversial effects on BCRP regulation by E2 on MCF-7 cells may be due to different E2 stimulus durations.

Furthermore, in a recent study where the ERα-negative human cell line MDA-MB-453 was transfected with plasmids containing *Bcrp* and *Erβ* genes, the treatment with E2 upregulated mRNA and protein BCRP expression. Tamoxifen reverted the effects induced by E2. In the same study, E2 also upregulated BCRP mRNA and protein expression in MBA-MB-468 cells. Also, in this cell line, the silencing of the *Erβ* gene prevented BCRP (mRNA and protein) upregulation by E2 [64]. Then, ERβ positively regulates BCRP expression. The treatment of the human placenta BeWo cells with estriol increased BCRP mRNA and protein expression, and ICI reverted these effects [141]. These data are concordant because estriol has a higher affinity for ERβ than ERα [39]. Another study reported that treatment of BeWo cells with E2 downregulated BCRP protein expression and ICI reverted the effect. Moreover, in the same study, it was shown that E2 in BeWo cells downregulates *Erβ* gene and does not affect *Erα* gene expression [140]. This can explain a possible E2 preference for ERα signaling in BeWo cells.

**Fig. 3:**
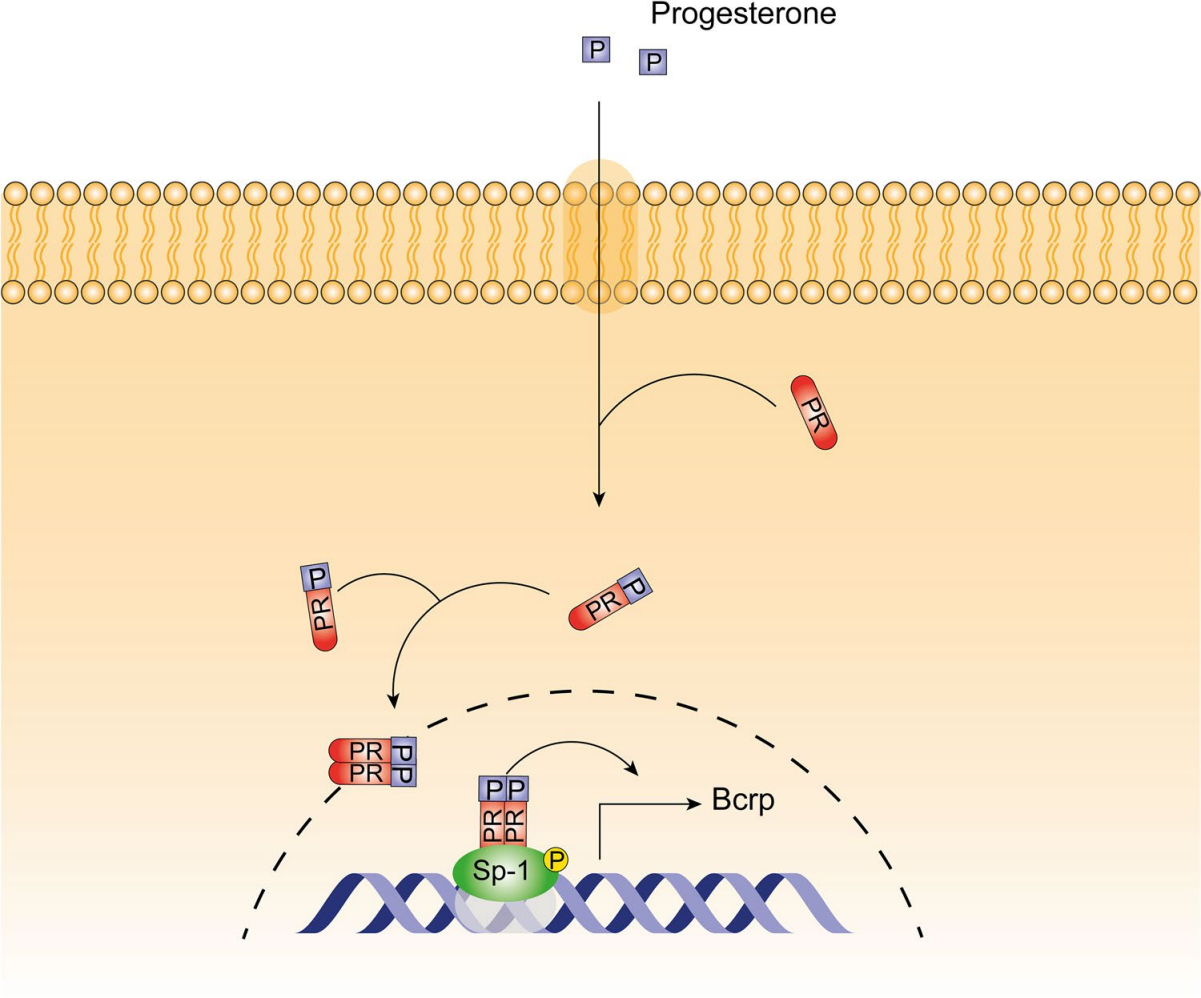
*Bcrp* regulation by progesterone. *Bcrp* gene transcription in regulated by progesterone through an indirect genomic signaling mechanism. Progesterone receptor (PR) promotes *Bcrp* gene transcription by interacting with Sp-1 transcription factor

P4 treatment of T47D cells transfected with a reporter plasmid containing a luciferase gene under a *Bcrp* gene promotor increased luciferase fluorescence. RU486, a PR antagonist, blocked the effects induced by P4. In addition, the treatment with P4 in the presence of mythramicin A, an Sp1 recruitment blocker, had no significant effect on luciferase fluorescence [146]. A co-immunoprecipitation of Sp1 and PR has already been reported, which indicates an interaction between the two proteins [95]. These data suggest that in T47D-cells, PR regulates *Bcrp* gene expression through an indirect genomic signaling mechanism. PR is recruited to *Bcrp* gene promotor by the interaction with Sp1 transcription factor (Fig. 3). The treatment of BeWo cells with P4 increased BCRP protein expression, but RU486 did not revert the effect induced by P4 [140]. This suggests a signaling through mPRs and highlights the hypothesis of a tissue-dependent regulation of BCRP by P4. In contrast, also in the BeWo cell line, the treatment with P4 downregulated *Bcrp* gene expression [145]. The treatment of BeWo cells with both P4 and E2 induced an upregulation in BCRP protein expression higher than with P4 alone. ICI and RU486 also reverted the cumulative effects induced by both sex steroid hormones [140].

**Fig. 4:**
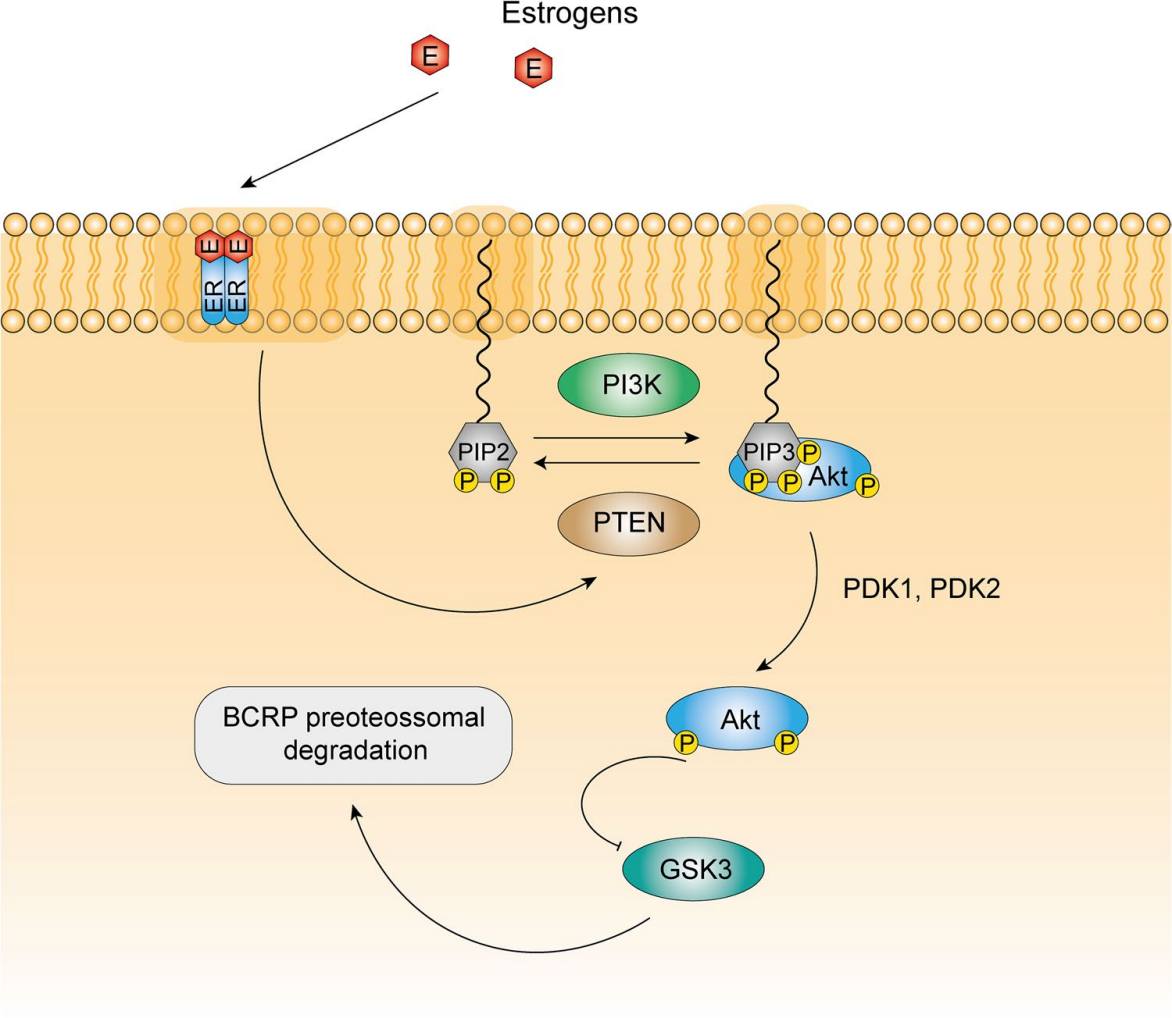
Hypothetical pathway of BCRP regulation by E2 in the brain capillary endothelial cells. The subpopulation of estrogen receptor in the plasma membrane may be responsible for the estrogens non-genomic effects. Upon activation ERβ leads to the activation of PTEN, which function as a negative Akt regulator through PIP3 dephosphorylation. This promotes the accumulation of GS3K in an active state (unphosphorylated) which may promote BCRP proteasomal degradation

In terms of signaling mechanisms, there are some studies that tried to uncover the signaling mechanism of BCRP regulation by estrogens in rat BBB. The treatment of isolated brain capillaries from female and male rats with E2 decreased BCRP (mRNA and protein) expression levels [42, 73]. Also, in vivo studies where mice were administrated with E2 showed a reduced expression of BCRP (protein) in brain capillaries [42]. Rat brain capillaries treated with E2 were incubated with BODIPY-Prazosin, a BCRP substrate, and the luminal fluorescence was measured by confocal microscopy. E2 treatment decreased BCRP activity in rat brain capillaries [41, 42, 73]. Similar results were obtained by treating rat brain endothelial cells with 17α-ethinylestradiol [91]. E2 treatment of brain capillaries isolated from ErαKO mice showed that E2 decreased BCRP protein expression and activity. However, the same treatment with E2 does not show significant effects in BCRP protein expression and activity in brain capillaries isolated from ERβKO mice [42, 73]. Rat brain capillaries were also incubated with selective agonists for ERα and ERβ, propyl pyrazole triol (PPT), and diarylpropionitrile (DNP), respectively. DNP decreased BCRP protein levels and activity in brain capillaries, while PPT did not significantly affect BCRP protein levels nor its activity. Rat brain capillaries were also treated with the ERα antagonist MPP or ICI. MPP did not reverte the downregulation of BCRP activity induced by E2, while ICI reverted the downregulation of BCRP protein expression and activity in rat brain capillaries when treated with E2 [73]. Then, in the BBB, BCRP is regulated by E2 through ERβ. Some contradictory results were also reported. Data showed a decrease in BCRP activity after the treatment of rat brain capillaries with PPT, and the treatment of ErβKO mice with E2 revealed significant effects in BCRP activity [41]. Further experiments tried to uncover the signaling mechanism by which E2 downregulated BCRP protein expression and activity. The inhibition of PI3K and Akt induced the downregulation of BCRP expression. This is the same effect observed with E2 treatment. Also, the inhibition of PTEN and GSK3 reverted the effects induced by E2. It was also demonstrated that E2 led to an increase in unphosphorylated PTEN, the activated form of the enzyme, and also led to a decrease in phosphorylated PTEN. For Akt, the contrary was observed. E2 led to an upregulation of inactive Akt and a downregulation of active Akt. Furthermore, the administration of lactacystin, a proteosome inhibitor, reverted the downregulation of BCRP induced by E2 [42]. Akt can inhibit GS3K by its phosphorylation [43], and GS3K phosphorylates proteins, signaling them for ubiquitination [105]. These results point to a putative signaling pathway that involves the activation of PTEN by E2, which leads to the downregulation of Akt and hence to an increase in active GS3K. Then, GS3K may lead BCRP to proteasome degradation (Fig. 4). These non-genomic effects possibly mediated by ERβ may be triggered by the subpopulation of ERs that lie in the plasma membrane [124].

The data about the regulation of BCRP by estrogens and progesterone suggests a tissue-dependent regulation of BCRP by these two hormones. The evidence concerning the regulation of BCRP by androgens is sparse and is mainly from studies with an animal model. In the human breast cancer cell line MCF-7, the treatment with DHT downregulated *Bcrp* gene expression levels [16]. In rodents, gonadectomy of male hamsters increased *Bcrp* gene expression in the Harderian gland [76]. Also, gonadectomy in male mice did not exert any effect on BCRP expression in the kidneys but downregulated *Bcrp* expression in the liver of male mice. DHT replacement increased *Bcrp* gene expression in the liver of gonadectomized male mice. Regarding female mice, gonadectomy increased *Bcrp* expression in the kidney and the administration of DHT increased *Bcrp* gene expression in the liver of ovariectomized female mice [134].

## Estrogens and its metabolic products as ABC drug transporter regulators

The most common estrogens are E2, E1, and estriol (E3). E2 and E1 can be interconverted by the action of 7β-hydroxysteroid dehydrogenase enzyme. On the other hand, E3, is synthetized through the hydroxylation of E2 or 16α-hydroxyestrone [113]. E1 and E3 are less active estrogens than E2 [152]. Stimulation of BeWo cells with E2 leads to the downregulation of BCRP [140, 141], and the stimulation with E3 leads to the upregulation [141]. These results probably arise from the fact that E3 elects the ERβ signaling pathway. The authors showed that the presence of ICI abrogated the effects induced by E2 and E3, and the knockdown of ERα did not revert the effects promoted by E3 [141]. This is in line with the evidence that E3 has a higher affinity for ERβ than for ERα [39].

MDR1 is able to transport E1 and E3 [54], and estrogens may possibly act as competitive inhibitor for the efflux of other substances. If the effect on transporter expression was neglected, estrogens may account for an easy drug absorption and distribution. In the case of the placenta, despite the downregulation of BCRP, E2 leads to an upregulation of MDR1. This is shown in JAR cells [20], a human placental cell line, and in human primary cytotrophoblasts [31]. There is also evidence of the *MRP1* gene upregulation by E1 in a placenta human cell line. In the placenta, MRP1 is located on the basolateral membrane of syncytiotrophoblasts [2]. In this case, E1could hypothetically contribute to a greater distribution of therapeutic drugs to the fetus. Although, like E3, E1 presents a lower affinity for ERs than E2 [9]. E2 is the principal urinary metabolite and in human renal proximal tubular epithelial cells is showed that E2 upregulates MDR1 [50]. This regulatory mechanism may be relevant in women due to the fluctuations of estrogens during the estrous cycle and pregnancy. E2 may act in order to balance the hypothetically increment in E3 (transported by MDR1) reabsorption as a consequence of the increase in plasma estrogens. A similar mechanism of compensation may not occur in the liver. Estradiol-17β-_D_-glucuronide is an estrogen metabolite which is able to be transported by MRP2 [35], but is also responsible for its internalization [6, 10, 153] being a cholestatic agent.

## Crosstalk between constitutive androstane receptor and sex steroids in the regulation of ABC drug transporters

Constitutive androstane receptor (CAR) acts as a xenobiotic and endobiotic receptor, capable of regulating the expression of genes involved in drug metabolism and transportation pathways, including ABC transporters. There are several studies showing that this receptor modulates the expression and activity of MDR1, MRP2, and BCRP [14, 63, 123, 142]. Swales and Negishi reviewed the influence of sex steroids on this receptor [131], reporting that P4 and androgens repress the transcriptional activity of CAR [52, 60] and pharmacological levels of E2, estrone and pregnane-3,20-dione, a P4 metabolite, are able to activate it [52, 86]. Thus, this constitutes an additional pathway for the regulation of ABC transporters by sex steroids. Other interesting point about CAR is that it influences the estrogen signaling by suppressing the p160 activators GRIP-1 and SRC-1, important for estrogenic signaling [80].

## Summary and concluding remarks

Men and women respond differently to drug treatments. Sex differences in drug efficacy as well as in the development of drug adverse reactions were reported. For example, women respond better than men to the antiarrhythmic verapamil [59]. Furthermore, women are more susceptible than men to generate adverse effects to morphine [108] and to the anti-tumor necrosis factor agent infliximab [148]. This diversity in drug effectiveness and adverse reactions probably arise from sex differences in pharmacokinetics and pharmacodynamics [33]. These differences are a result of sex-specific factors like bodyweight, organ size, percentage of body fat, plasma proteins, metabolizing enzymes, and drug transporters [133]. These differences are well reviewed in the following references [71, 125, 133]. For instance, women show a higher CYP3A4 activity than man [147]. CYP3A4 is the most predominant enzyme of CYP3A family involved in the metabolism of almost 50% of the available drugs [126]. The regulation by sex hormones may be one of the keys to the differences in those factors. As mentioned by Madla et al., postmenopausal and premenopausal women have a different response to antidepressants [57]. During menopause, the level of circulating estrogens drops, suggesting the involvement of sex steroids in drug response. This highlights the importance of knowing the sex differences and the influence of sex hormone in drug pharmacokinetics and pharmacodynamics for the future development of personalized therapies.

ABC transporters are capable of extruding multiple drugs out of cells and play a major role in pharmacoresistance. Drugs of basically all classes are substrates of ABC transporters [35]. The collected evidence shows that MDR1, MRP1, MRP3, MRP4, MRP5, and BCRP have a sex different expression in several tissues or cell lines (summarized in Table 1). The gathered data also demonstrate that ABC transporters enumerated before are regulated by sex hormones (summarized in Table 2). The sex differences described for ABC transporters were determined in terms of mRNA and protein expression and were reported from studies in animal models. The evidence about the effects of the sex hormones in ABC drug transporters activity is also scarce. In fact, only few studies have accessed the effects of sex hormones in ABC drug transporters activity in human cells. Despite the lack of functional studies, there is no doubt that ABC transporters are regulated by sex hormones. Taking into consideration that we do not know exactly whether sex hormones influence the ABC transporters activity in human cells, neither if there are functional differences between sexes, nor if that putative differences in the functioning of ABC drug transporters arise from sex hormones, it is imperative to perform more studies and move further on this topic.

The collected data showed a tissue-dependent regulation of ABC transporters by sex hormones. This is may be due to the differential expression of sex hormone receptors in those tissues. As previously said, besides ABC transporters, many factors, like plasma proteins and metabolizing enzymes, are able to influence the efficacy of a drug in a sex dependent manner [71, 125]. Each of these factors are just a piece of the puzzle and knowing the regulation of them by sex steroids is needed for a further characterization of the molecular mechanisms that are behind the sex differences in response to a drug treatment. As a final remark for the development and optimization of therapeutic strategies accounting on sex differences, more investigations are needed, and further studies on the regulation of ABC transporters, as well as on other molecules involved in pharmacokinetics and pharmacodynamics, by sex steroids, will need to be undertaken.
